# Natural Killer Cells for Cancer Immunotherapy: Pluripotent Stem Cells-Derived NK Cells as an Immunotherapeutic Perspective

**DOI:** 10.3389/fimmu.2014.00439

**Published:** 2014-09-15

**Authors:** Cristina Eguizabal, Olatz Zenarruzabeitia, Jorge Monge, Silvia Santos, Miguel Angel Vesga, Natalia Maruri, Arantza Arrieta, Marta Riñón, Estibaliz Tamayo-Orbegozo, Laura Amo, Susana Larrucea, Francisco Borrego

**Affiliations:** ^1^Basque Center for Transfusion and Human Tissues, Galdakao, Spain; ^2^Immunopathology Group, BioCruces Health Research Institute, Barakaldo, Spain; ^3^Regulation of the Immune System Group, BioCruces Health Research Institute, Barakaldo, Spain; ^4^Ikerbasque, Basque Foundation for Science, Bilbao, Spain

**Keywords:** NK cells, adoptive cell therapy, cancer immunotherapy, hematopoietic stem cell transplantation, pluripotent stem cells, embryonic stem cells, induced pluripotent stem cells

## Abstract

Natural killer (NK) cells play an essential role in the fight against tumor development. Over the last years, the progress made in the NK-cell biology field and in deciphering how NK-cell function is regulated, is driving efforts to utilize NK-cell-based immunotherapy as a promising approach for the treatment of malignant diseases. Therapies involving NK cells may be accomplished by activating and expanding endogenous NK cells by means of cytokine treatment or by transferring exogenous cells by adoptive cell therapy and/or by hematopoietic stem cell transplantation. NK cells that are suitable for adoptive cell therapy can be derived from different sources, including *ex vivo* expansion of autologous NK cells, unstimulated or expanded allogeneic NK cells from peripheral blood, derived from CD34+ hematopoietic progenitors from peripheral blood and umbilical cord blood, and NK-cell lines. Besides, genetically modified NK cells expressing chimeric antigen receptors or cytokines genes may also have a relevant future as therapeutic tools. Recently, it has been described the derivation of large numbers of functional and mature NK cells from pluripotent stem cells, both embryonic stem cells and induced pluripotent stem cells, which adds another tool to the expanding NK-cell-based cancer immunotherapy arsenal.

## Introduction

Natural killer (NK) cells are innate lymphoid cells that have an important role in regulating the defenses to viral infections and cancer development (1–6). The vast majority of circulating mature human NK cells in healthy donors are identified as CD3−CD56+ lymphocytes. Approximately, 90% of peripheral blood and spleen NK cells belong to the CD56^dim^CD16^+^ subset, which is characterized by a potent cytotoxic activity after interaction with target cells. On the other hand, NK cells on lymph nodes and tonsils are mostly CD56^bright^CD16^dim/−^ and have poor cytotoxic activity, while they produce very significant amounts of cytokines, such as interferon (IFN)-γ, in response to IL-12, IL-15, IL-18, and type I IFN stimulation (7, 8). NK cells are equipped with an array of activating and inhibitory receptors that stimulate or dampen NK-cell activity, respectively. Inhibitory receptors include the MHC class I ligands killer-cell immunoglobulin-like receptors (KIRs) with two or three extracellular immunoglobulin domains and long cytoplasmic tail (KIR2DL and KIR3DL), leukocyte immunoglobulin-like receptor subfamily member 1 (LILRB1) and CD94/NKG2A, and other inhibitory receptors such as CD300a, leukocyte-associated immunoglobulin-like receptor-1 (LAIR-1), and others. Activating receptors include cytokine and chemokine receptors, and those that interact with ligands expressed on target cells. The latter include, among others, the natural cytotoxicity receptors or NCRs (NKp30, NKp44, and NKp46), NKG2D, KIR with short cytoplasmic tail (KIR2DS and KIR3DS), CD94/NKG2C, CD244, and DNAM-1. In addition, NK cells also express the death ligands FasL and TRAIL that after interaction with death receptors Fas and DR5, respectively, initiate a signaling cascade resulting in apoptosis of the target cell. Finally, NK cells express FcγRIIIA or CD16, the receptor that exerts antibody-dependent cell-mediated cytotoxicity (ADCC) (4, 9–13).

Natural killer-cell effector functions are dynamically regulated, and the killing or sparing of target cells depends on the integration of distinct signals that emanate from NK-cell receptors after their interaction with ligands expressed on target cells. NK cells spare healthy cells that express MHC class I molecules and low amounts of stress-induced self-molecules, while they kill target cells that up-regulate stress-induced self-molecules and/or down-regulate MHC class I molecules (4, 5, 11, 12). The latter are common features of virus-infected cells and tumors (14, 15). The investigation of NK-cell reactivity has revealed the basis of tumor recognition, and several lines of evidence have shown that NK cells have a critical role in host immunity against cancer (2, 16–19). In response, tumors have evolved mechanisms to escape control from NK cells, such as the modulation of NK-cell receptor–ligand expression patterns and the secretion of immunoregulatory molecules or immunosuppressive modulators such as IDO, PGE2, and TGF-β, that down-regulate NK-cell effector functions (20–24).

So far, all the amassed knowledge has driven efforts to harness NK cells with the purpose to improve the therapeutic options for patients living with cancer. Indeed, NK-cell-based adoptive cell immunotherapy is emerging as a promising approach for treatment of many cancers (25–27). Therapeutic NK cells can be derived from different sources, including peripheral blood or cord blood cells, adult hematopoietic stem cells (HSCs), embryonic stem cells (ESCs), or induced pluripotent stem cells (iPSCs).

## NK-Cell-Based Immunotherapy

Given the role that NK cells have in the defense against tumor development, the therapeutic use of NK cells to treat malignancies is currently being exploited. It is very well established that NK cells have a very important role in the anti-tumor effect of therapeutic antibodies that use ADCC as a mechanism of action (28–31). In addition, in the clinical context, several approaches have been proposed for NK-cell-based immunotherapy, including *in vivo* cytokine-mediated expansion of endogenous NK cells, as well as the adoptive transfer of unmodified or *ex vivo* activated and expanded autologous and allogeneic NK cells, and some NK-cell lines, such as NK-92 (26, 32–41). Furthermore, genetically modified NK cells expressing cytokine genes or chimeric antigen receptor (CAR), are being studied for potential use in the clinic (26, 42–44). In clinical trials, NK-cell infusions alone or in the course of allogeneic hematopoietic stem cell transplantation (HSCT), are being tested as therapy for refractory tumors. In addition, they are also tested as consolidation immunotherapy, which could be an important therapeutic tool in high risk hematological malignancies during the remission phase after chemotherapy, and when allogeneic HSCT is not indicated due to its high degree of toxicity (45, 46).

Early studies were aimed to *in vivo* expand endogenous NK cells and to improve their anti-tumor activity by administering systemic cytokines, such as IL-2, into patients (47–49). Other strategies included the *ex vivo* activation and expansion of autologous NK cells, following their adoptive transfer into the patients in combination with IL-2 (32, 50–53). These approaches offered poor clinical outcomes due to high toxicity of IL-2 (54). Moreover, this cytokine promoted the expansion not only of NK cells but also of regulatory T (Treg) cells, therefore dampening NK cells effector functions (55). Others have assessed the effects of low-dose IL-2 administration and IL-2 boluses on NK-cell activation after autologous HSCT (39, 56). Whereas IL-2 significantly expanded the number of circulating NK cells *in vivo*, these NK cells did not exhibit maximal cytotoxic potential as determined by *in vitro* assays (39). In addition, although the infusion of IL-2-activated NK-cell-enriched populations or intravenous IL-2 infusions combined with subcutaneous IL-2 augmented *in vivo* the NK-cell function, there was a lack of consistent clinical efficacy of autologous NK-cell-based therapy in patients with lymphoma and breast cancer when compared with cohorts of matched controls (56).

Although relatively safe, the lack of significant efficacy of therapy with autologous NK cells could be due to the interaction of MHC class I molecules expressed on cancer cells that, after their interaction with MHC class I-specific inhibitory receptors on NK cells, suppress their activation (4, 10–12). Specifically, since human NK cells are regulated by KIRs that interact with specific HLA class I molecules, it is expected that in HLA-non-identical transplantation where the recipients lack the class I epitope specific for the donor’s inhibitory KIRs (i.e., receptor–ligand mismatch), donor NK cells will be not inhibited, leading to a better prognosis due to a decreased risk of relapse. In fact, clinical data have shown that haploidentical KIR ligand-mismatched NK cells play a very important role as anti-leukemia effector cells in the haploidentical T cell-depleted transplantation settings (57, 58). Several publications have revealed that patients with acute myeloid leukemia (AML) are significantly more protected against leukemia relapse when they receive a transplant from NK alloreactive donors (38, 57–62). Furthermore, several strategies using adoptively transferred allogeneic NK cells have been shown to be successful for cancer immunotherapy, including those against leukemia and solid tumors (36, 63–66). Table 1 depicts a summary of completed clinical trials that have used infusion of allogeneic NK cells. Importantly, the infusion of allogeneic NK cells has also been demonstrated to be a safe therapy with low toxicity (38). Prominently, there are also clinical studies that have confirmed that infusion of donor–recipient inhibitory KIR-HLA-mismatched NK cells, following mild conditioning, is well tolerated by pediatric patients, which indicates that this is a promising novel therapy for reducing the risk of relapse in children with tumors (45, 67).

**Table 1:** **Selected completed clinical trials that have used infusion of allogeneic NK cells in https://clinicaltrials.gov**.

Indication	Cell product	Combined	Center (country)	Clinicaltrials. gov identifier
Advanced cancer	NK cells	Allogeneic HSCT	Asan Medical Center (Korea)	NCT00823524
AML	IL-2 activated NK cells	Chemotherapy, IL-2, and denileukin diftitox	Masonic Cancer Center, University of Minnesota (USA)	NCT01106950
AML	NK cells	Chemotherapy and IL-2	St. Jude Children’s Research Hospital (USA)	NCT00187096
AML	IL-2 activated NK cells	Chemotherapy and IL-2	Masonic Cancer Center, University of Minnesota (USA)	NCT00274846
AML	UCB NK cells	Chemotherapy, IL-2, TBI, and UCB transplant	Masonic Cancer Center, University of Minnesota (USA)	NCT00871689
AML and MDS	IL-2 activated NK cells	Chemotherapy, IL-2, and allogeneic HSCT	M.D. Anderson Cancer Center (USA)	NCT00402558
Breast cancer	IL-2 activated NK cells	Chemotherapy, IL-2, and TBI	Masonic Cancer Center, University of Minnesota (USA)	NCT00376805
Hematological malignancies	UCB NK cells	IL-2, TBI, UCB transplantation	Masonic Cancer Center, University of Minnesota (USA)	NCT00354172
Hematological malignancies	NK cells	Autologous HSCT	Tufts Medical Center (USA)	NCT00660166
Hematological malignancies	NK cells	Rituximab, Rhu-GMCSF, and allogeneic HSCT	M.D. Anderson Cancer Center (USA)	NCT00383994
Hematological malignancies	NK cells	Haploidentical HSCT	Asan Medical Center (Korea)	NCT00569283
Hematological malignancies	NK cells	Allogeneic-matched HSCT	Duke University Medical Center (USA)	NCT00586690
Hematological malignancies	NK cells	Allogeneic-mismatched HSCT	Duke University Medical Center (USA)	NCT00586703
Lymphoma and solid tumors	IL-2 expanded with irradiated autologous feeder cells		Seoul National University Hospital (Korea)	NCT01212341
Melanoma	NK cells	Chemotherapy and IL-2	Seoul National University Hospital (Korea)	NCT00846833
Multiple myeloma	NK cells	Chemotherapy, IL-2, and autologous HSCT	University of Arkansas (USA)	NCT00089453
NHL or CLL	IL-2 activated NK cells	Rituximab, IL-2, and chemotherapy	Masonic Cancer Center, University of Minnesota (USA)	NCT00625729
Non-B lineage hematologic malignancies and solid tumors	Expanded NK cells	Chemotherapy and IL-2	St. Jude Children’s Research Hospital (USA)	NCT00640796
Ovarian, fallopian tube, and primary peritoneal cancer	IL-2 activated NK cells	Chemotherapy, IL-2, and TBI	Masonic Cancer Center, University of Minnesota (USA)	NCT00652899
Ovarian, fallopian tube, peritoneal, and breast cancer	IL-2 activated NK cells	Chemotherapy and IL-2	Masonic Cancer Center, University of Minnesota (USA)	NCT01105650
Poor prognosis non-AML hematologic malignancies	NK cells	Chemotherapy and IL-2	St. Jude Children’s Research Hospital (USA)	NCT00697671
Solid tumors	IL-15 activated NK cells	Haploidentical HSCT	Hospital Infantil Universitario Niño Jesús (Spain)	NCT01337544

*AML, acute myeloid leukemia; CLL, chronic lymphocytic leukemia; HSCT, hematopoietic stem cell transplantation; MDS, myelodysplastic syndrome; NHL, non-Hodgkin lymphoma; TBI, total body irradiation; UCB, umbilical cord blood*.

Using NK-cell lines as source for the treatment of cancer may also be beneficial. Specifically, the use of NK-92 cell line has been demonstrated to be a safe therapy with anti-tumor effects (41, 68, 69). In fact, the FDA has approved the testing of NK-92 infusions in patients with advanced solid tumors (68).

The successful use in the clinic of CAR-expressing T cells in the treatment of hematological malignancies has prompted the development of other CAR-expressing cytotoxic cells. In this context, preclinical studies are being carried out investigating the targeting of tumors using CAR-redirected NK cells (43, 70–79). Although the majority of these studies have been performed against targets of hematological origin, it has also been described as promising results with NK cells transduced with CARs specific for antigens expressed on solid tumors (75, 78, 79). Mostly, all these studies have been done with the NK-92 cell line transduced with the specific CAR, although *in vitro* stimulated NK cells from healthy donors and pediatric leukemia patients have also been used (70).

In order to successfully use NK-cell infusions in the clinical setting, a sufficient number of highly enriched NK cells must be obtained. Allogeneic unmodified NK cells can be adoptively transferred after leukapheresis products are T cell-depleted, in combination with B cell depletion and/or NK-cell enrichment (67, 80, 81). In the context of allogeneic HSCT, the transfer of unmodified NK cells or CD3/CD19-depleted grafts results in recovery of elevated NK-cell numbers, which can also expand *in vivo* (67, 81, 82). In the absence of HSCT, successful NK-cell expansion *in vivo* is achieved by the administration of IL-2 in combination with products that deplete Treg cells (80).

Various methods for large-scale and clinical-grade *ex vivo* NK-cell expansion have been reported with this aim (83–92). Due to the advantage of aseptic conditions in a closed system, peripheral blood mononuclear cells (PBMCs) collected by leukapheresis are frequently used as source for goods manufacturing practice (GMP)-compliant expansion of NK cells (84, 85, 87). In general, the expansion of allogeneic NK cells involves two sequential steps. The first consists in the magnetic depletion of CD3+ T lymphocytes, followed by a second step of enrichment of CD56+ NK cells (83, 85, 87, 90). To expand the purified NK cells, they are cultured with cytokines, such as IL-2, IL-12, IL-15, and IL-21 (84, 85, 87, 93, 94). In order to further encourage NK-cell proliferation, several authors have used irradiated feeder cells in the culture, such as PBMCs, Epstein–Barr virus-transformed lymphoblastoid cell lines or engineered leukemic cell lines (83, 86, 90, 95). Irradiated feeder cells stimulate NK cells through both humoral factors and direct cell-to-cell contact. However, there are technical disadvantages by using supportive feeder cell lines that could lead to problems with the regulatory agencies.

CD34+ hematopoietic progenitors from umbilical cord blood (UCB) are also being considered as a source for the production of a large number of allogeneic NK cells (89, 91, 92, 96, 97). Some groups have described different protocols for the generation of NK cells from CD34+ cells using coculture systems with stromal cell lines and a combination of cytokines that promote the development of NK cells (88, 97, 98). Very importantly, other authors have been able to generate large numbers of UCB CD34+ cells-derived NK-cell products for adoptive immunotherapy in closed, large-scale bioreactors, and stromal cell lines free, for the use in future clinical trials (91, 92). These NK cells have been shown to efficiently target bone marrow-residing human leukemia cells in preclinical studies (96). It is important to investigate, which cytokines added to these cultures favors the generation of higher numbers of mature NK cells with enhanced effector functions. For example, it has been shown that IL-12 directs human NK-cell differentiation *ex vivo* from CD34+ cord blood precursors toward more mature NK cells with improved properties (93).

Obtaining a significant number of pure and functional NK cells is a critical factor for NK-cell-based immunotherapy. Several authors have shown the efficient generation of a large number of functional and mature NK cells from human embryonic stem cells (hESCs) and iPSCs, suggesting that the clinical use of these NK cells may be a reasonable expectation for the future of cancer immunotherapy (99–104).

## Pluripotent Stem Cells: ESCs and iPSCs

Since the derivation of hESCs, more than 20 years ago by Thomson et al., numerous groups have successfully differentiated these cells into fully mature and functional cells from each germ layer (105). Shortly, after the original derivation of hESCs, various groups demonstrated the hematopoietic development using an *in vitro* model and defined conditions (103, 104, 106–111).

One of the scientific breakthroughs of the last years has been to determine that pluripotency can be recovered by several differentiated somatic cell types through the overexpression of just four transcription factors (OCT4, SOX2, cMYC, and KLF4) (112–114). These cells are named iPSCs. Depending on the donor’s somatic cell type, the reprograming process is accomplished with different efficiency. Just 7–12 days are required to reprogram mouse embryonic fibroblasts (MEFs) (115), whereas human foreskin fibroblasts take 20–25 days, using retrovirus technology in both cases (116). Compared with fibroblasts, human keratinocytes can be reprogramed 100 times more efficiently and twofold faster (116). After choosing the target donor somatic cell type, it is necessary to select a cocktail of reprograming factors that usually are the four above mentioned. In few situations less than four factors are needed, such as in the case of cord blood CD133+ cells and keratinocytes (117). Through the reprograming process, the chromatin remodeling plays an essential role in the procurement of pluripotency. So far, it has been described that the use of some chemical compounds is able to alter the DNA methylation and induce chromatin remodeling that results in an improvement of the reprograming process. For example, treatment with DNA methytransferase inhibitor (5′-azacytidine) and histone deacetylase inhibitors (SAHA, TSA, and VPA) improves reprograming efficiency in MEFs. Also, during the reprograming process, it is important to maintain the pluripotency state. This can be achieved by using compounds that inhibit glycogen synthase kinase 3, lysine-specific demethylase 1, or G9a (118–122). Once iPSCs are generated, they have the capability to differentiate toward ectodermal, mesodermal, endodermal, and germ cells. This is achieved by the addition to the culture media of some growth factors and several compounds that provide specific signals allowing iPSCs to differentiate in the cell type of interest (123).

Another important issue during the reprograming process is the method for the delivery of the transcription factors into the somatic cells. Currently, there are integrative delivery systems (retrovirus, lentivirus, linear DNA, and piggyBac transposon) and non-integrative systems (adenovirus, Sendai viral vectors, episomal vectors, synthetic mRNA, and proteins) (123, 124). The choice of one or another system will depend on the final use of the human-induced pluripotent stem cells (hiPSCs). For research purposes, the usual methods are the integrative systems, whereas if hiPSCs are intended for future clinical use, the non-integrative methods should be more appropriated.

### Generation of NK cells from hESCs and hiPSCs

**Figure 1:**
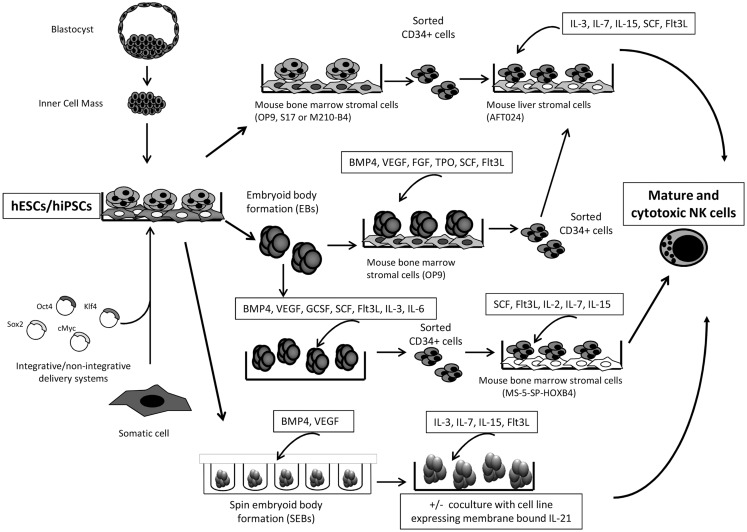
**Schematic representation for the generation of human ESC/iPSC-derived NK cells is shown**. Summary of several protocols described in Ref. (100, 101, 104, 111, 129–132).

Pluripotent stem cells (PSCs) are an important advance in stem cell research, as they allow researchers to obtain stem cells, which, in addition to be very useful tools for research, they may have therapeutic uses. Because hiPSCs are developed from a patient’s own somatic cells, it is believed that hiPSCs-based therapy would be very poorly or non-immunogenic, whereas hESCs are not (125–128). The use of these cells provides an accessible, genetically tractable, and homogenous starting cell population to efficiently study human blood cell development among others (100, 103, 108, 111, 129). hESCs and hiPSCs can provide important starting cell populations to develop new cell-based therapies that have the potential to treat both malignant and non-malignant diseases. The clinical applications of this type of cell-based therapy depend on the thoroughly understanding of the normal development and physiology of the PSCs and of the desired “final” cell population. Several groups have already demonstrated the ability of hESC and hiPSC-derived hematopoietic progenitor cells to produce functional NK cells that, hypothetically at least, could serve as a “universal” source of anti-tumor lymphocytes for cancer immunotherapy (99–104, 130, 131) (Figure 1). In addition, hiPSCs, which can be reliably engineered *in vitro*, provide an important new model system to study human NK-cell development, as well as a model for NK-cell deficiency and diseases with significant defects on NK-cell functions (108).

Generating CD34+ hematopoietic precursors is the first important step in the specific hematopoietic lineage differentiating protocols from hESCs and hiPSCs. The initial protocols achieved to obtain up to 20% of CD34+ cells by coculturing the hESCs with the OP9 mouse bone marrow stromal cells (111). Other groups obtained similar results using the S17 or M210-B4 mouse bone marrow stromal cell lines, and they were able to *in vitro* generate CD34+CD45− and CD34+CD45+ precursors (104, 131, 132). It has been described that hESCs-derived CD34+CD45+ cells contain more hematopoietic progenitors, and consequently are more suitable for the NK-cell differentiation when compared with the CD34+CD45− population (104). Usually, after the generation of hESCs- and hiPSCs-derived CD34+ hematopoietic precursors, these are sorted and subsequently cultured under conditions that favor the development of NK cells. For example, sorted hESCs-derived CD34+ cells were placed in culture with the murine fetal liver-derived AFT024 stromal cell line as feeder cells in medium supplemented with IL-15, IL-3, IL-7, SCF, and fms-like tyrosine kinase receptor-3 ligand (Flt3L) (104). At the end of the culture process, after 30 days, NK cells expressed maturation markers including KIRs, CD94/NKG2A, NCRs, and CD16 (104). In addition, these cells could lyse malignant cells by both direct cell-mediated cytotoxicity and ADCC. On the other hand, Knorr et al. have also proved the trafficking of hESC-derived NK cells to K562 tumor cells engrafted in sublethally irradiated mice for 4 days before NK-cell injection (130).

Other approach for the generation of CD34+ hematopoietic precursors *in vitro* is to differentiate both types of PSCs by embryoid body (EB) assays followed by a coculture system with the OP9 stromal cell line and a cocktail of cytokines, such as BMP4, VEGF, SCF, FGF, TPO, and Flt3L (129, 133). EBs are three-dimensional aggregates of PSCs, which resembles the embryonic development, including the differentiation toward cells of the hematopoietic lineage. Knorr and colleagues have used a refined method of the EBs assay, termed spin EBs, in the presence of BMP4 and VEGF and, after a period of 11 days of spin EB differentiation, they add IL-3, IL-7, IL-15, and Flt3L, that favors the development of NK cells (100, 130).

Other important factor for the *in vitro* differentiation of NK cells from PSCs is the role of the HOXB4 homeoprotein. Larbi et al. have described that HOXB4 delivery promotes the enrichment and expansion of EB-derived hematopoietic precursors that could differentiate into fully mature and functional NK cells (101). HOXB4 protein, in combination with stromal cells, has an important role in the development of NK cells from hESCs, suggesting the potential use of this protein for NK-cell enrichment from PSCs.

A step forward is the clinical-scale production of NK cells derived from PSCs for future cancer immunotherapy applications. Kaufman’s group has improved the method for the clinical-scale generation of NK cells. They used a two-stage culture system to efficiently generate NK cells from hESCs and iPSCs in the absence of cell sorting and without the need for xenogeneic stromal cells. As mentioned above, the method is based on the combination of spin EB formation using defined conditions and membrane-bound interleukin 21-expressing artificial antigen-presenting cells that allow the production of mature and functional NK cells from several different hESC and iPSC lines. They are able to generate enough cytotoxic and mature NK cells to treat a single patient starting from fewer than 250,000 input hESCs/iPSCs that could be maintained and continuously expanded for at least 2 months (100).

## Future Directions

Adoptive immunotherapy with NK-cell infusions is currently used in patients with high risk of relapse after HSCT (34, 38, 67). Even though preliminary results are encouraging, still critical issues remain unanswered, such as the characterization of standardized protocols for GMP-compliant production of clinical-grade NK cells. Apart from that, with continued advances in the stem cell field, it is likely that hPSC-derived NK cells will relatively soon be able to be efficiently derived on a patient-specific basis. Actually, hESC and hiPSC-derived NK cells express activating and inhibitory receptors similar to NK cells isolated from adult peripheral blood (100, 104, 108, 130). The hESC-derived NK cells are also highly efficient at direct cell-mediated cytotoxicity and ADCC, as well as cytokine (IFN-γ) production. And importantly, stromal cells-free protocols have successfully been described (100, 130). It is clear that hiPSC-derived NK cells provide a genetically manageable system to study human NK-cell development and function. In addition, these NK cells could provide an important source of lymphocytes for cancer therapy. There are several and serious obstacles to be overcome before PSC-derived NK cells can be considered for cancer immunotherapy. Safe methods for hiPSC generation and high reprograming efficacy are of the highest importance. Furthermore, the irreversible nature of hPSC-based therapy requires special precautions to be taken in any clinical trial. We have to be realistic and accept that multiple technical, safety, and regulatory obstacles are in the way for successful translation of hPSC-derived NK cells into the clinic. But hopefully, in a not so far future, all these hurdles will be surmounted and the use of hPSCs-based cancer therapies will be a reality.

## Conflict of Interest Statement

The authors declare that the research was conducted in the absence of any commercial or financial relationships that could be construed as a potential conflict of interest.
